# Synergic value of 3D CT-derived body composition and triglyceride glucose body mass for survival prognostic modeling in unresectable pancreatic cancer

**DOI:** 10.3389/fnut.2025.1499188

**Published:** 2025-03-19

**Authors:** Kangjing Xu, Xinbo Wang, Changsheng Zhou, Junbo Zuo, Chenghao Zeng, Pinwen Zhou, Li Zhang, Xuejin Gao, Xinying Wang

**Affiliations:** ^1^Department of General Surgery, Jinling Hospital, Affiliated Hospital of Medical School, Nanjing University, Nanjing, China; ^2^Department of Radiology, Jinling Hospital, Affiliated Hospital of Medical School, Nanjing University, Nanjing, China; ^3^Department of General Surgery, The Affiliated People’s Hospital of Jiangsu University, Zhenjiang, China

**Keywords:** unresectable pancreatic cancer, body composition, skeletal muscle index, triglyceride glucose-body mass index (TyG-BMI), 3D CT reconstruction, survival prognosis

## Abstract

**Background:**

Personalized and accurate survival risk prognostication remains a significant challenge in advanced pancreatic ductal adenocarcinoma (PDAC), despite extensive research on prognostic and predictive markers. Patients with PDAC are prone to muscle loss, fat consumption, and malnutrition, which is associated with inferior outcomes. This study investigated the use of three-dimensional (3D) anthropometric parameters derived from computed tomography (CT) scans and triglyceride glucose-body mass index (TyG-BMI) in relation to overall survival (OS) outcomes in advanced PDAC patients. Additionally, a predictive model for 1 year OS was developed based on body components and hematological indicators.

**Methods:**

A retrospective analysis was conducted on 303 patients with locally advanced PDAC or synchronous metastases undergoing first-line chemotherapy, all of whom had undergone pretreatment abdomen-pelvis CT scans. Automatic 3D measurements of subcutaneous and visceral fat volume, skeletal muscle volume, and skeletal muscle density (SMD) were assessed at the L3 vertebral level by an artificial intelligence assisted diagnosis system (HY Medical). Various indicators including TyG-BMI, nutritional indicators [geriatric nutritional risk index (GNRI) and prealbumin], and inflammation indicators [(C-reactive protein (CRP) and neutrophil to lymphocyte ratio (NLR)] were also recorded. All patients underwent follow-up for at least 1 year and a dynamic nomogram for personalized survival prediction was constructed.

**Results:**

We included 211 advanced PDAC patients [mean (standard deviation) age, 63.4 ± 11.2 years; 89 women (42.2) %)]. Factors such as low skeletal muscle index (SMI) (*P* = 0.011), high visceral to subcutaneous adipose tissue area ratio (VSR) (*P* < 0.001), high visceral fat index (VFI) (*P* < 0.001), low TyG-BMI (*P* = 0.004), and low prealbumin (*P* = 0.001) were identified as independent risk factors associated with 1 year OS. The area under the curve of the established dynamic nomogram was 0.846 and the calibration curve showed good consistency. High-risk patients (> 211.9 points calculated using the nomogram) had significantly reduced survival rates.

**Conclusion:**

In this study, the proposed nomogram model (with web-based tool) enabled individualized prognostication of OS and could help to guide risk-adapted nutritional treatment for patients with unresectable PDAC or synchronous metastases.

## Introduction

Pancreatic ductal adenocarcinoma (PDAC) is one of the most frequent cause of cancer-associated mortality (1), with 80%–85% of patients diagnosed at an advanced or metastatic stage, having no opportunity for curative resection (2, 3). Despite advancements in systemic chemotherapy over the past years, the prognosis of patients with unresectable or metastatic PDAC remains dismal, with a 5 years survival rate less than 3% (4). Prognostic factors are, therefore, essential, and accurate identification of poor prognostic factors may help guide more aggressive treatment protocols for individuals with advanced (locally extensive vascular involvement and metastatic) PDAC.

The physiologic effects of PDAC can dramatically weaken patients, wasting their skeletal muscle mass and adipose tissue (5). The wasting syndrome of cachexia is present in up to 80% of PDAC patients at diagnosis, who exhibit poor treatment tolerance and resistance to many antineoplastic therapies, coupled with rapid disease progression and low rates of pathologic complete response even with the most effective systemic agents (6). Although body mass index (BMI) provides a general insight into patient mass, it fails to elucidate body composition nuances, particularly the distribution disparities between metabolically active visceral fat and inert subcutaneous fat (7–9). In this regard, recent studies have highlighted the prognostic value of computed tomography (CT) image-based markers of body composition in cancer prognosis, showing that low psoas and skeletal muscle indices (PMI and SMI) are associated with inferior survival outcomes in colorectal, esophageal and lung cancer (10–12). In parallel, CT-derived skeletal muscle density (SMD) acts as a marker for myosteatosis and complements SMI in quantifying muscle function in addition to volume, in line with the contemporary European Working Group on Sarcopenia in Older People 2 (EWGSOP2) definition (13–15). Furthermore, the visceral fat is significantly proinflammatory and has been demonstrated to be an indicator associated with poor prognosis in gastric, rectal, and breast cancers (16–18). Previous studies calculating the body composition were based on a two-dimensional (2D) basis by segmentation of CT sectional images at the level of the L3 vertebra, which is only an approximation of the actual body composition and lacks accuracy. Moreover, this approach depended to varying degrees on manual processing, which is time- and labor-intensive and does not align with integration in routine care. Consequently, body composition analysis is still largely neglected in survival risk stratification. The advent of deep learning and image segmentation technologies has enabled automated, three-dimensional (3D) CT reconstruction for precise body composition analysis, allowing more granular and accurate prediction of prognosis (19, 20).

Moreover, PDAC typically induces metabolic reprogramming, triggering systemic metabolic disorders such as abnormal glucose tolerance and insulin resistance (IR), pivotal factors closely related to tumor proliferation, metastasis and cachexia (21). A nationwide cohort study demonstrated compared with the metabolic syndrome-free group, the metabolic syndrome-persistent group had the higher risk of pancreatic cancer (22). Triglyceride glucose-body mass index (TyG-BMI), which is calculated based on triglyceride (TG), fasting plasma glucose (FPG), and BMI, has been recognized as a powerful and simple tool to assess IR (23). Studies have demonstrated that the TyG-BMI index has a substantial correlation with a number of illnesses, including non-alcoholic fatty liver disease, diabetes mellitus, and coronary heart disease (24–26). Nevertheless, the current literature lacks investigation regarding its prognostic implications in PDAC. Since both pancreatic cancer and the TyG index are associated with metabolic syndrome, we hypothesized that there would be a direct association between pancreatic cancer and the TyG index. Thus, in this study, we included the TyG index in analyses.

Against this background, our study endeavors to explore the anthropometric features evaluated through CT scans utilizing an automatic 3D deep learning software and to analyze prognostic factors in unresectable or metastatic PDAC patients undergoing at least 3 cycles of first-line chemotherapy. The study further aimed to develop a novel prediction model of 1 year overall survival (OS) based on independent risk factors, encompassing differential body compositions, metabolic indicators and nutritional parameters, with the goal of integration into routine clinical practice to enable personalization.

## Materials and methods

### Study design

**FIGURE 1:**
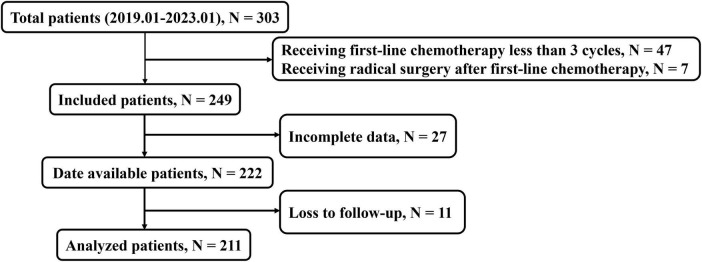
Flowchart depicting the process of patient enrolment.

We retrospectively evaluated 303 patients with unresectable (due to extensive vascular involvement or organ invasion) or metastatic PDAC treated at Jinling Hospital or the Affiliated People’s Hospital of Jiangsu University, including 211 patients treated with first-line chemotherapy at least three cycles in the final analysis (Figure 1) between January 2019 and January 2023. The OS time was defined as the duration from the initiation of chemotherapy treatment to the date of death from any cause. We excluded patients for receiving first-line chemotherapy fewer than three cycles (*n* = 47), receiving radical resection surgery after chemotherapy (*n* = 7), incomplete data (*n* = 27), or loss to follow-up (*n* = 11). The first-line chemotherapy regimens included FOLFIRINOX (folinic acid, fluorouracil, irinotecan, and oxaliplatin), gemcitabine based treatments [AG (gemcitabine plus nab-paclitaxel), GS (gemcitabine plus S-1)] and AS (nab-paclitaxel plus S-1) treatment. The study was approved by the Ethics Committee of Jinling Hospital (No. 2023DZKY-049-03) and registered at ClinicalTrials.gov (registration ID: NCT06378853).

### CT anthropometric measurements

**FIGURE 2:**
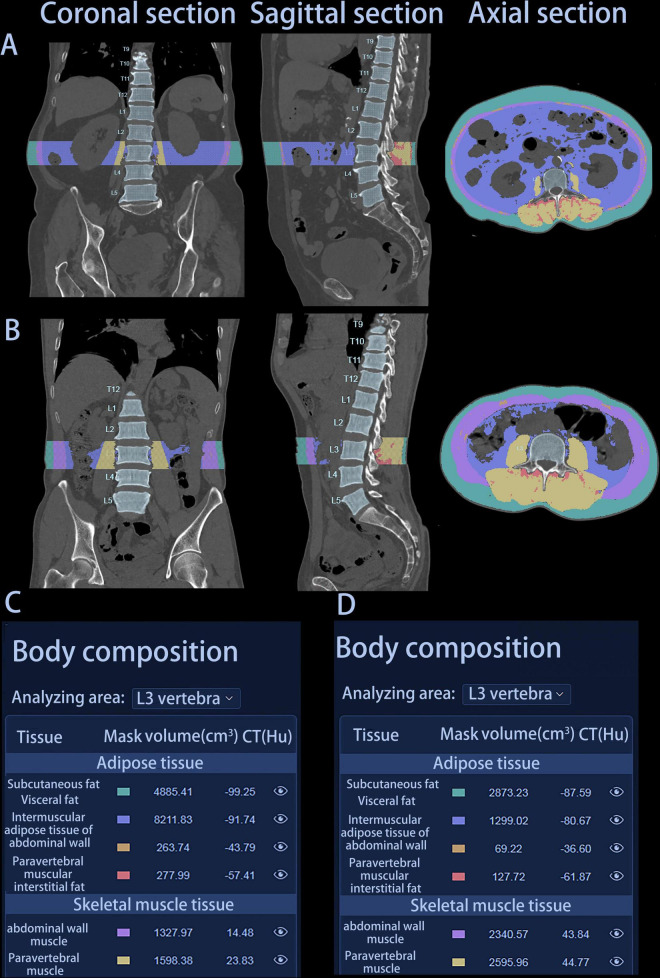
Graphical representation of two patients’ automatic report generated by HY Medical. **(A)** Coronal, sagittal, and axial section of patient A. **(B)** Coronal, sagittal, and axial section of patient B. **(C)** Body composition parameters of patient A. **(D)** Body composition parameters of patient B. Patient A: female, 75 years, survival time 142 days; Patient B: male, 76 years, survival time 407 days.

Height and weight measurements obtained prior to treatment were used to calculate BMI. The HY Medical system, an intelligent quantitative CT assisted diagnosis tool, was employed to extract body composition parameters from CT images. This software can automatically measure 3D volume and CT Hounsfield unit (HU) of various anatomical components, such as the abdominal wall muscle, paravertebral muscle, subcutaneous fat, visceral fat, intermuscular adipose tissue of abdominal wall and paravertebral muscular interstitial fat at the L3 vertebra level in cubic centimeters on multislice CT scans in less than 60 s (Figure 2). This software conducts deep learning-based segmentation of fat (visceral and subcutaneous) and muscle voxels. Additionally, we obtained calculated variables based on the information of these parameters. The skeletal muscle volume (cm^3^) was calculated by summing the volumes of the abdominal wall muscle and paravertebral muscle. The total fat body volume (cm^3^) was the sum of the subcutaneous fat volume, visceral fat volume, the volume of intermuscular adipose tissue of abdominal wall, and paravertebral muscular interstitial fat volume. The intermuscular adipose tissue volume (IMAT, cm^3^) was obtained by adding the volume of intermuscular adipose tissue of abdominal wall to the volume of paravertebral muscular interstitial fat. SMD = (HU of the abdominal wall muscle × abdominal wall muscle volume + HU of the paravertebral muscle × paravertebral muscle volume)/(abdominal wall muscle volume + paravertebral muscle volume). The SMI (cm^3^/m^2^), total fat index (TFI) (cm^3^/m^2^), subcutaneous fat index (SFI) (cm^3^/m^2^) and visceral fat index (VFI) (cm^3^/m^2^) were calculated by normalizing skeletal muscle volume, total fat body volume, subcutaneous fat volume and visceral fat volume to the height, respectively.

### Data collection

Pretreatment baseline levels of lymphocytes, monocytes, neutrophils, C-reactive protein (CRP), hemoglobin, albumin, globulin, prealbumin, FBG, TG and creatinine were measured by laboratory examination. Additionally, we obtained calculated variables based on the information of these variables, namely, geriatric nutritional risk index (GNRI), albumin to globulin ratio (AGR), TyG-BMI, neutrophil to lymphocyte ratio (NLR) and lymphocyte to monocyte ratio (LMR). The GNRI was calculated using the formula: GNRI = 14.89 × serum albumin (g/dL) + 41.7 × [present body weight (kg)/ideal body weight (kg)]. TyG-BMI was calculated using the formula: TyG-BMI = ln [TG (mg/dL) × FBG (mg/dL)/2] × BMI.

### Definition of cut-off values

Receiver operating characteristic (ROC) analysis was performed to assess the predictive value of various factors, including BMI, SMI, SMD, IMAT, TFI, VFI, SFI, visceral to subcutaneous adipose tissue area ratio (VSR), GNRI, AGR, TyG-BMI, NLR, CRP, LMR, and creatinine on 1 year OS, selected as a clinically relevant endpoint. The optimal cut-off value was determined by maximizing specificity and sensitivity using Youden’s Index. Low hemoglobin levels were defined as < 120 g/L for male patients and < 110 g/L for female patients based on local laboratory standards.

### Statistical analysis

All statistical analysis were performed using IBM SPSS Statistics for Windows, version 25.0 (IBM Corp., Armonk, NY, United States) and R version 4.3.2 (R Project for Statistical Computing, Vienna, Austria). Continuous variables were tested for normality, and quantitative data were described as means with standard deviations (SD) (normal distribution) or medians with quartile spacing (non-normal distribution). Categorical variables were described as numbers and proportions. Spearman’s correlation coefficient was utilized to evaluate correlations between the different body composition parameters and hematological indicators. The Kaplan–Meier method and log-rank tests were used to estimate and compare the 1 year OS rates between the two groups. Univariate and multivariate survival analyses were performed using the Cox’s proportional hazards model to identify prognostic factors. Clinical variables with a significance level of *P* < 0.05 in the univariate analysis were incorporated in the multivariate COX regression using both forward- and backward-likelihood ratio methods. Furthermore, a dynamic nomogram for survival prediction was formulated using predictive factors and internally validated using 1,000 bootstraps. The performance of the nomogram was assessed using the C-index, the area under the time-dependent ROC curve (time ROC-AUC), a calibration curve and decision curve analysis (DCA) for the entire study cohort. The total points were calculated for each patient based on the established nomogram, and three groups of patients with different risks of prognosis (based on the total points) were delineated using the X-tile program (27). Survival curves were plotted among these three groups using the Kaplan–Meier method. All *P*-values were two-sided, and *P* < 0.05 was considered statistically significant.

## Results

### Baseline patient characteristics

After applying stringent exclusions, a total of 211 patients with unresectable or metastatic PDAC were included in this retrospective study. Table 1 displays the baseline demographic characteristics of the population in the study cohort. The mean (SD) age was 63.4 (11.2) years, and the majority of patients were men (57.8%). Among these patients, 76 patients (36.0%) were diagnosed at the locally advanced (unresectable) stage due to extensive vascular involvement, typically the superior mesenteric vessels. Of the remaining 135 cases (64.0%) with distant metastasis, 77 had liver metastasis, 19 had lung metastasis, 31 had abdominal metastasis, and eight had bone metastasis. Ninety-seven patients received FOLFIRINOX, 84 patients received gemcitabine-based chemotherapy and 30 received AS chemotherapy.

**TABLE 1:** Characteristics of the population.

Characteristic	Total patients (*N* = 211)
**Gender, N (%)**	
Male	122 (57.8)
Female	89 (42.2)
Age, years	63.4 ± 11.2
**Diabetes mellitus, N (%)**	
Yes	66 (31.3)
No	145 (68.7)
BMI, kg/m^2^	22.0 ± 3.2
**Tumor status, N (%)**	
Invading surrounding organs or blood vessels	76 (36.0)
Liver metastasis	77 (36.5)
Other (lung, abdominal and bone metastasis)	58 (27.5)
**First-line chemotherapy, N (%)**	
FOLFIRINOX	97 (46.0)
Gemcitabine based	84 (39.8)
AS	30 (14.2)
SMI, cm^3^/m^2^	2238.1 ± 532.0
SMD, HU	38.4 ± 8.7
IMAT, cm^3^	244.3 (174.7–335.8)
TFI, cm^3^/m^2^	4759.2 (3591.9–6202.3)
SFI, cm^3^/m^2^	2068.0 (1396.7–2809.3)
VFI, cm^3^/m^2^	2518.7 (1749.8–3245.2)
VSR	1.2 (0.9–1.7)
Hemoglobin, g/L	122.6 ± 17.6
Prealbumin, mg/L	197.2 ± 60.0
GNRI	101.0 ± 10.5
AGR	1.7 ± 0.5
TyG-BMI	197.1 ± 36.1
NLR	2.8 (2.0–4.0)
CRP, mg/L	2.7 (0.6–10.9)
LMR	3.0 (2.1–4.2)
Creatinine, μmol/L	53.0 (44.9–62.8)

BMI, body mass index; SMI, skeletal muscle index; SMD, skeletal muscle density; IMAT, intermuscular adipose tissue volume; TFI, total fat index; SFI, subcutaneous fat index; VFI, visceral fat index; VSR, visceral to subcutaneous adipose tissue area ratio; GNRI, geriatric nutritional risk index; AGR, albumin to globulin ratio; TyG-BMI, triglyceride glucose-body mass index; NLR, neutrophil to lymphocyte ratio; CRP, C-reactive protein; LMR, lymphocyte to monocyte ratio.

### Variables and their correlations

**FIGURE 3:**
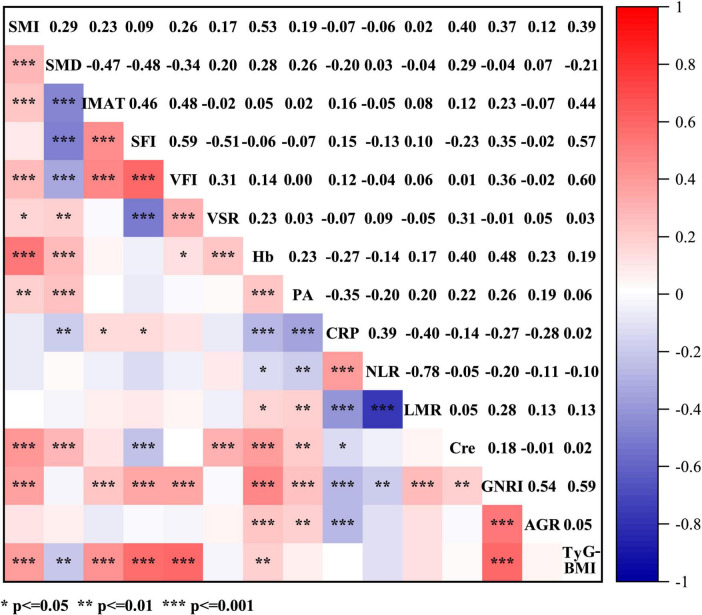
Spearman’s correlations between anthropometric parameters (SMI, SMD, IMAT, SFI, VFI, and VSR) and hematological indicators (Hb, PA, CRP, NLR, LMR, Cre, GNRI, AGR, and TyG-BMI). SMI, skeletal muscle index; SMD, skeletal muscle density; IMAT, intermuscular adipose tissue volume; TFI, total fat index; SFI, subcutaneous fat index; VFI, visceral fat index; VSR, visceral to subcutaneous adipose tissue area ratio; Hb, hemoglobin; PA, prealbumin; CRP, C-reactive protein; NLR, neutrophil to lymphocyte ratio; LMR, lymphocyte to monocyte ratio; Cre, creatinine; GNRI, geriatric nutritional risk index; AGR, albumin to globulin ratio; TyG-BMI, triglyceride glucose-body mass index.

Concerning the body composition parameters and their correlations, as visible in the correlogram (Figure 3), SFI exhibited a positive correlation with VFI (ρ = 0.59), while SMD displayed negative correlations with IMAT, SFI and VFI (ρ = −0.47, ρ = −0.48 and ρ = −0.34, respectively). Among the hematological indicators, hemoglobin, prealbumin, GNRI and AGR can reflect the nutritional status, and immunological status of patients with cancer as it was defined by NLR, CRP and LMR (28, 29). In regards of correlations among these variables, GNRI showed positive correlations with AGR and TyG-BMI (ρ = 0.54 and ρ = 0.59, respectively), and CRP exhibited inverse correlations with prealbumin and LMR (ρ = −0.35 and ρ = −0.4, respectively). As for associations among body composition parameters and hematological indicators, SMI was highly correlated with hemoglobin and TyG-BMI (ρ = 0.53 and ρ = 0.39, respectively), while SFI displayed high correlations with GNRI and TyG-BMI (ρ = 0.35 and ρ = 0.57, respectively). Additionally, SMD was positively associated with prealbumin (ρ = 0.26) and inversely associated with CRP (ρ = −0.20).

### ROC curve and survival analysis

**FIGURE 4:**
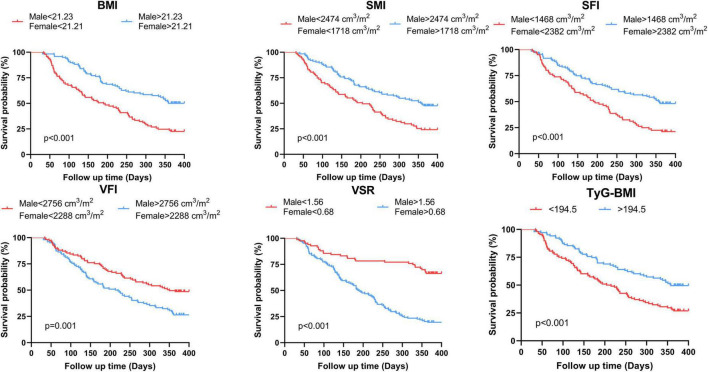
Kaplan-Meier estimates of overall survival according to the threshold determined on ROC analysis of BMI, SMI, SFI, VFI, VSR and TyG-BMI for the whole population. BMI, body mass index; SMI, skeletal muscle index; SFI, subcutaneous fat index; VFI, visceral fat index; VSR, visceral to subcutaneous adipose tissue area ratio; TyG-BMI, triglyceride glucose-body mass index.

The analysis of ROC curves for the anthropometric parameters and hematological indicators in relation to OS is summarized in Table 2. With the exception of SMD (AUC = 0.57, *p* = 0.080), IMAT (male: AUC = 0.57, *p* = 0.163; female: AUC = 0.51, *p* = 0.844), and TFI (male: AUC = 0.53, *p* = 0.557; female: AUC = 0.55, *p* = 0.467), all body composition parameters are statistically significant. Among the hematological indicators, only TyG-BMI (AUC = 0.60, *p* = 0.012) provided prognostic value for 1 year survival. Figure 4 and Supplementary Figure 1 illustrate Kaplan-Meier analysis with log-rank tests using cut-offs determined from ROC curve analysis for BMI, SMI, SFI, VFI, VSR, and TyG-BMI. All parameters were identified as significant risk factors for OS (*p* < 0.01), considering cut-off values of 21.23 kg/m^2^ for males and 21.21 kg/m^2^ for females for BMI, 2474.00 cm^3^/m^2^ for males and 1718.00 cm^3^/m^2^ for females for SMI, 1468.00 cm^3^/m^2^ for males and 2382.00 cm^3^/m^2^ for females for SFI, 2756.00 cm^3^/m^2^ for males and 2288.00 cm^3^/m^2^ for females for VFI, 1.56 for males and 0.68 for females for VSR, and 194.50 for TyG-BMI. When these thresholds were applied to the patient cohort, there were 93 patients (44.08%) with low BMI, 87 patients (41.23%) with low SMI, 80 patients (37.91%) with low SFI, 102 patients (48.34%) with high VFI, 128 patients (60.66%) with high VSR, and 108 patients (51.18%) with low TyG-BMI.

**TABLE 2:** Diagnostic performance of three-dimensional (3D) anthropometric parameters measured on computed tomography (CT) and hematological indicators for 1 year overall survival using an receiver operating characteristic (ROC) analysis.

	Cut-offs	AUC	*P*-value
BMI, kg/m^2^	–	–	–
Male/female	21.23/21.21	0.63/0.66	**0.018/0.013**
SMI, cm^3^/m^2^	–	–	–
Male/female	2474.00/1718.00	0.61/0.62	**0.045/**0.064
SMD, HU	38.89	0.57	0.080
IMAT, cm^3^	–	–	–
Male/female	252.50/367.80	0.57/0.51	0.163/0.844
TFI, cm^3^/m^2^	–	–	–
Male/female	3682.00/5003.00	0.53/0.55	0.557/0.467
SFI, cm^3^/m^2^	–	–	–
Male/female	1468.00/2382.00	0.62/0.59	**0.028/**0.185
VFI, cm^3^/m^2^	–	–	–
Male/female	2756.00/2288.00	0.54/0.68	0.441/**0.004**
VSR	–	–	–
Male/female	1.56/0.68	0.77/0.75	**< 0.001/< 0.001**
GNRI	101.30	0.57	0.098
AGR	1.63	0.51	0.847
TyG-BMI	194.50	0.60	**0.012**
NLR	3.45	0.58	0.059
CRP, mg/L	11.48	0.54	0.296
LMR	2.63	0.53	0.401
Creatinine, μmol/L	55.6 ± 18.1	–	–
Male/female	60.90/38.50	0.54/0.55	0.470/0.444

Bold values indicate *p* < 0.05. BMI, body mass index; SMI, skeletal muscle index; SMD, skeletal muscle density; IMAT, intermuscular adipose tissue volume; TFI, total fat index; SFI, subcutaneous fat index; VFI, visceral fat index; VSR, visceral to subcutaneous adipose tissue area ratio; GNRI, geriatric nutritional risk index; AGR, albumin to globulin ratio; TyG-BMI, triglyceride glucose-body mass index; NLR, Neutrophil to lymphocyte ratio; CRP, C-reactive protein; LMR, lymphocyte to monocyte ratio.

The median follow-up time was 313 [interquartile range (IQR), 128–387] days, and 131 patients (62.09%) died within 1 year of treatment. Notably, 1 year OS in the low BMI, low SMI, low SFI and low TyG-BMI groups was significantly shorter (log-rank *P* < 0.001) than in the high groups (Figure 4). Nonetheless, there were noticeable survival advantages for low VFI and low VSR compared to the high groups (*P* = 0.001, *P* < 0.001).

### Cox analysis

**FIGURE 5:**
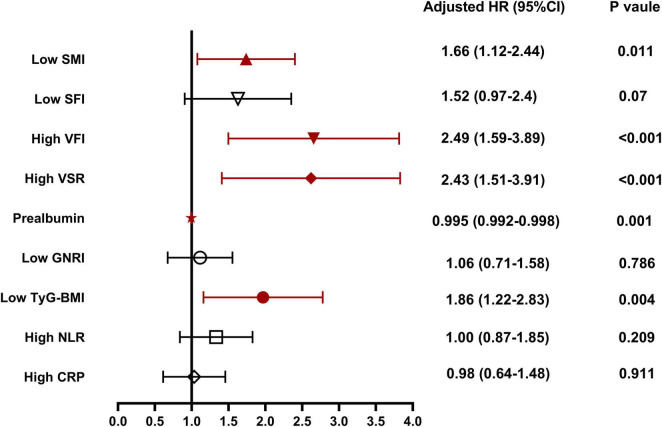
Independent risk factors for 1 year overall survival for patients with unresectable or metastatic pancreatic ductal adenocarcinoma (PDAC). SMI, skeletal muscle index; SFI, subcutaneous fat index; VFI, visceral fat index; VSR, visceral to subcutaneous adipose tissue area ratio; GNRI, geriatric nutritional risk index; AGR, albumin to globulin ratio; TyG-BMI, triglyceride glucose-body mass index; NLR, neutrophil to lymphocyte ratio; CRP, C-reactive protein.

Table 3 presents the results of Cox analysis. In the univariate analysis, body composition parameters such as low SMI, low SFI, high VFI, and high VSR, low nutritional indicators like prealbumin and GNRI, low metabolic indicator TyG-BMI, and high inflammation indicators including NLR and CRP, were significantly associated with inferior survival in patients with unresectable or metastatic PDAC. In the multivariable analysis, low SMI [hazard ratios (HR), 1.66; 95% confidence interval (CI), 1.12–2.44; *P* = 0.011)], high VFI (HR, 2.49; 95% CI, 1.59–3.89; *P* < 0.001), high VSR (HR, 2.43; 95% CI, 1.51–3.91; *P* < 0.001), prealbumin (HR, 0.995; 95% CI, 0.992–0.998; *P* = 0.001), and low TyG-BMI (HR, 1.86; 95% CI, 1.22–2.83; *P* = 0.004) emerged as independent predictors of 1 year OS when both forward-LR and backward-LR methods were used, as shown in the forest plot (Figure 5).

**TABLE 3:** Univariate analysis of risk factors for 1 year mortality in the whole cohort.

	*P*-value	HR (95% CI)
Gender	0.460	0.89 (0.62–1.24)
Age	0.069	1.01 (1.00–1.03)
Tumor status	–	–
Invading surrounding organs or blood vessels	Reference	–
Liver metastasis	0.753	1.07 (0.72–1.59)
Other (lung, abdominal and bone metastasis)	0.935	1.02 (0.66–1.58)
First-line chemotherapy	–	–
FOLFIRINOX	Reference	–
Gemcitabine based	0.079	0.72 (0.49–1.04)
AS	0.359	0.79 (0.47–1.31)
Low SMI	**< 0.001**	1.89 (1.34–2.66)
Low SMD	0.075	1.34 (0.97–1.96)
Low IMAT	0.126	1.33 (0.92–1.92)
Low TFI	0.377	1.17 (0.82–1.67)
Low SFI	**< 0.001**	2.01 (1.42–2.84)
High VFI	**0.002**	1.74 (1.23–2.46)
High VSR	**< 0.001**	3.65 (2.39–5.57)
Low hemoglobin	0.690	1.08 (0.74–1.59)
Prealbumin	**< 0.001**	0.995 (0.992–0.998)
Low GNRI	**0.022**	1.50 (1.06–2.12)
High AGR	0.114	1.32 (0.94–1.86)
Low TyG-BMI	**< 0.001**	1.86 (1.31–2.63)
High NLR	**0.005**	1.65 (1.16–2.34)
High CRP	**0.016**	1.60 (1.09–2.34)
Low LMR	0.069	1.38 (0.98–1.95)
High creatinine	0.664	1.08 (0.76–1.53)

Bold values indicate *p* < 0.05. SMI, skeletal muscle index; SMD, skeletal muscle density; IMAT, intermuscular adipose tissue volume; TFI, total fat index; SFI, subcutaneous fat index; VFI, visceral fat index; VSR, visceral to subcutaneous adipose tissue area ratio; GNRI, geriatric nutritional risk index; AGR, albumin to globulin ratio; TyG-BMI, triglyceride glucose-body mass index; NLR, neutrophil to lymphocyte ratio; CRP, C-reactive protein; LMR, lymphocyte to monocyte ratio.

### Nomogram construction and validation

**FIGURE 6:**
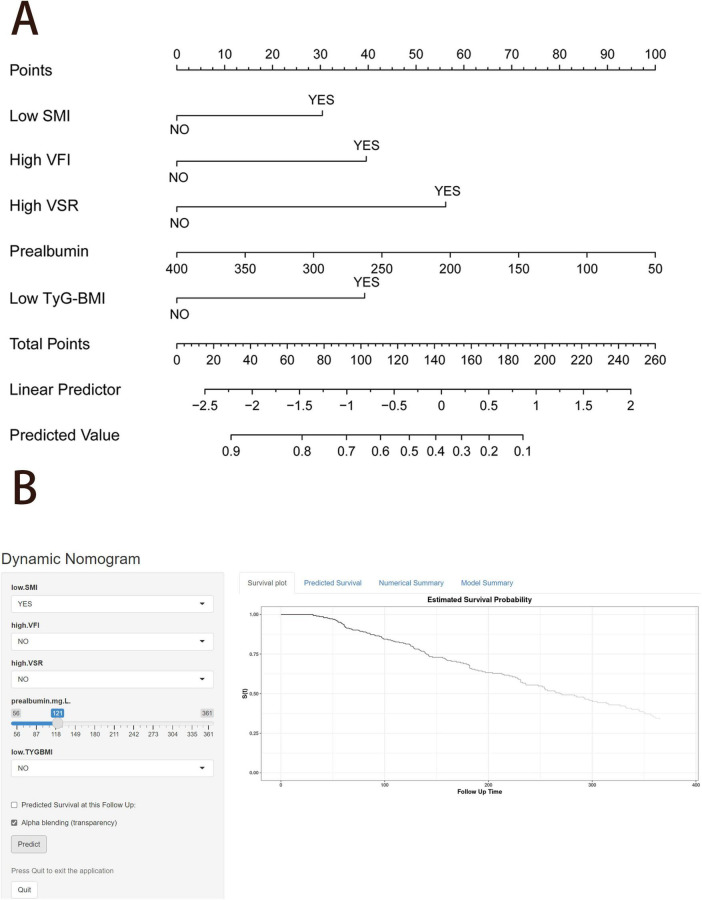
Nomogram to predict the probability of 1 year overall survival. **(A)** Nomogram for predicting 1 year overall survival for patients with unresectable or metastatic pancreatic ductal adenocarcinoma (PDAC). **(B)** An example diagram on the dynamic nomogram. SMI, skeletal muscle index; SFI, subcutaneous fat index; VFI, visceral fat index; VSR, visceral to subcutaneous adipose tissue area ratio; TyG-BMI, triglyceride glucose-body mass index.

**FIGURE 7:**
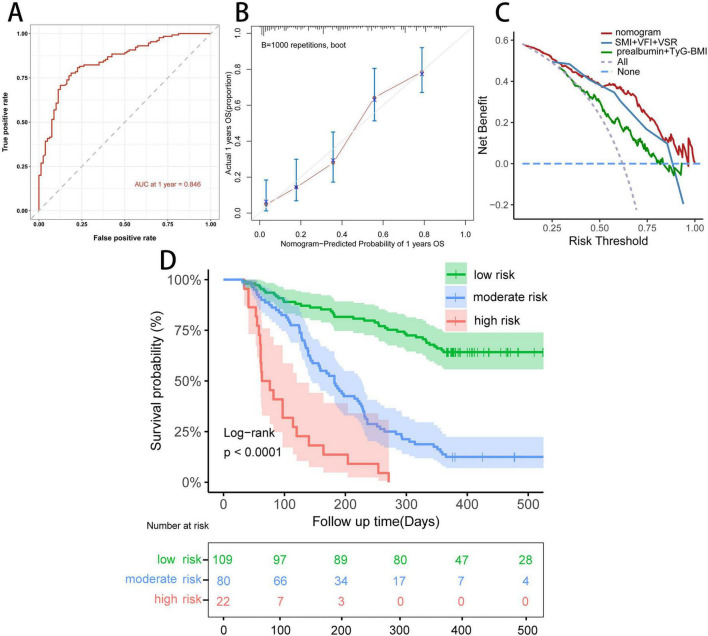
Evaluation of the predictive ability of the nomogram. **(A)** Time-dependent ROC curve of the nomogram at 1 year in the study cohort. **(B)** Calibration curve of the nomogram at 1 year in the study cohort. **(C)** Decision curve analysis for 1 year overall survival in the study cohort. **(D)** Kaplan–Meier curve of the nomogram model.

We constructed a nomogram integrating the five prognostic factors to predict the probability of 1 year OS based on the Cox model (Figure 6A). In the time-ROC analysis, the nomogram showed good discriminative ability in identifying the 1 year OS of patients with unresectable or metastatic PDAC, with an AUC of 0.846 (95% CI, 0.79–0.90; Figure 7A). Internal validation and calibration of the nomogram were conducted using 1,000 bootstrap analyses. The model yielded a C-index of 0.728, and after bootstrapping, the C-index was 0.719. The calibration plot of the nomogram (Figure 7B) revealed a strong correlation between the observed and predicted 1 year OS rates. DCA of the nomogram is shown in Figure 7C, showing that if the threshold probability of 1 year mortality was between 50% and 80%, utilizing the nomogram for predicting 1 year OS provided more benefit than relying solely on body compositions or hematological indicators alone.

We further stratified patients with unresectable or metastatic PDAC into three risk groups: low (< 147.5 points), middle (147.5 ≤ total point ≤ 211.9 points), and high (> 211.9 points) to evaluate the subgroups of patients positively influenced by the nomogram. The Kaplan–Meier curves for the study cohort (Figure 7D) indicated that the nomogram provided effective prognostic classification for the probability of 1 year OS (log-rank *P* < 0.001) in patients with unresectable or metastatic PDAC.

### Web-based calculator

Finally, we created a user-friendly predictive dynamic nomogram for OS using the independent predictors demonstrated in Figure 6B, which is now accessible online^1^. For patients with unresectable or metastatic PDAC, upon entering the values of the five variables into the dynamic nomogram, the anticipated time for “Time_death” is provided by selecting the “Predicted Survival at this Follow Up” option. Subsequently, by selecting the “Alpha blending (transparency)” option and clicking “Predict,” users can view the survival plot, the predicted survival, and the numerical summary of the patient.

## Discussion

This study investigated the prognostic value of anthropometric parameters, nutritional indicators, TyG-BMI, combined with inflammation indicators in a large population of patients with unresectable PDAC or synchronous metastasis treated with first-line chemotherapy. Most previously published studies were limited because they focused primarily on the impact of only one or two indicators on survival outcomes in PDAC patients. For instance, one study analyzing body compositions of 456 patients reported that high SMD and SFI were significantly associated with improved OS in patients with metastatic pancreatic cancer (mPC) (30). Similarly, Sema Turker et al showed that high NLR independently predicted adverse OS in mPC (31). In our investigation, body composition parameters including SMI, VFI, and VSR, nutritional indicator prealbumin, and metabolic indicator TyG-BMI were significant predictors of survival in patients with unresectable or metastatic PDAC. Low SMI, low prealbumin, and low TyG-BMI were linked to inferior 1 year OS, while low VFI and low VSR correlated with superior 1 year OS. Furthermore, a simple and practical dynamic nomogram was established using the above independent risk factors, which accurately predicted 1 year OS and properly identified the high-risk population (> 211.9 points).

Sarcopenia was originally defined as an age-related decline in skeletal muscle but has since been expanded to reflect the negative effects of low muscle mass on physical performance and clinical outcomes in a wide range of disease states, beyond the older population (32). A meta-analysis of 5,965 patients with esophageal cancer (EC) from 41 studies revealed a significant association between sarcopenia and poor survival (HR, 1.68, 95%CI, 1.54–1.83, *P* = 0.004) of EC patients (33). Similar results were observed in another systematic review and meta-analysis, where sarcopenic patients tended to have a significantly shorter 1 year OS rate (HR, 1.35, 95%CI, 1.08–1.68) (34). Our results concerning SMI are consistent with those observed in the literature (35). From a pathophysiological standpoint, this observation could be explained by a vicious circle of chronic inflammation and malnutrition leading to cachexia and sarcopenia in cancer patients, thereby affecting immune system and response to chemotherapy (36).

Today, adipose tissue is recognized as a kind of secretory organ that produces proinflammatory and anti-inflammatory cytokines and adipokines (37). Interestingly, subcutaneous fat proposed to possess predominantly anti-inflammatory as opposed to proinflammatory properties (38). High visceral fat is significantly proinflammatory and has been demonstrated to be an indicator associated with poor prognosis in various cancers. This phenomenon may explain why BMI is not a reliable prognostic marker in cancer and cancer surgery, as it fails to reflect differential fat distribution or muscle mass distribution (39). Some basic studies on the specific mechanism of visceral fat and tumor have confirmed a positive correlation between visceral adipose tissue and angiogenic biomarkers, particularly with circulating pro-angiogenic biomarker vascular endothelial growth factor A (VEGFA) levels (40). These may be possible mechanisms for the poor prognosis of patients with high VFI. However, in clinical studies of cancer patients, the effects of visceral adipose tissue on prognosis still ambiguous. Yang et al found that high VFI was significantly associated with better OS and PFS in cancer patients (41). Also, Zhou et al reported that high VFI had significantly improved OS than low VFI in hepatocellular carcinoma (42). However, Deng et al reported that high VFI was confirmed as a positive independent prognostic factor for OS and PFS in esophageal cancer, whose conclusion was consistent with ours (43). In the future, the exact predictive role of VFI needs to be explored in more cancer types and patients. Our study is the first to focus on pretreatment VSR, an indicator of body fat distribution, particularly the difference in visceral fat and subcutaneous fat levels, in patients with unresectable or metastatic PDAC, and investigate its impact on 1 year OS. Consistent with findings from studies involving patients with different cancers, high VSR was significantly associated with poor outcomes compared with high total body fat or visceral fat levels alone (44). In our study, most patients with high VSR are mainly due to low SFI. Therefore, strengthening the nutritional management to increase SFI, and reducing VSR may effectively improve the prognosis of unresectable PDAC patients.

Recently, there has been a gradual increase in the use of 3D rather than 2D segmentation to assess body composition on CT scans, resulting in a total volume measurement instead of mere area measurement at the L3 abdominal level (45). Fully automated 3D CT scan could more accurately identify muscle tissue and adipose tissue, enabling a more accurate and precise measurement of different parts of muscle and adipose tissue, especially for IMAT and SMD, compared to 2D segmentation computed (46, 47). Additionally, 3D analysis offers the advantages of time efficiency and high consistency, thus routine use could be envisaged in the future. For example, these measurements could be taken automatically during diagnostic or follow-up CT scan and recorded in the patient’s report, making them readily available to any clinician who wished to use it and reducing deviations associated with manual analyzing.

Triglyceride glucose-body mass index has superiority in assessing IR in both patients with and without diabetes and applicability to all subjects regardless of their status as recipients of insulin treatment. However, many studies have presented inconsistent opinions regarding the prognostic impact of TyG-BMI in different diseases. Some studies suggest that TyG-BMI may serve as a simple and solid index for assessing the risk of major adverse cardiovascular events (MACEs), with higher TyG-BMI index related to an increased incidence of MACEs (24, 48). Conversely, other studies propose an inverse association between TyG-BMI and all-cause mortality, for example, in critically ill patients with atrial fibrillation and in patients with heart failure (23, 49). Nevertheless, there is a paucity of previous studies on the association between TyG-BMI and cancer, especially in PDAC patients. TyG-BMI and mortality from all causes in patients with unresectable or metastatic PDAC were investigated for the first time in this study, revealing a strong inverse association between the two variables. Further clinical trials are warranted to explore the connection between TyG-BMI and patients with unresectable or metastatic PDAC.

While our study identified several body composition parameters, TyG-BMI, and prealbumin that were significantly associated with survival in patients with unresectable or metastatic PDAC treated by first-line chemotherapy, it is important to consider the potential limitations of these findings. Firstly, we studied morphological parameters before treatment and did not perform follow-up measurements to assess their prognostic value. Initial and longitudinal changes in body composition with a 3D measurement should be studied on a larger population. Moreover, although we collected data on pretreatment skeletal muscle mass, we did not collect data on grip strength or physical activity, which are key diagnostic factors for sarcopenia to comprehensively evaluate muscle quality according to the latest European Working Group on Sarcopenia in Older People (15). In addition, our study was retrospective in design and lacked external validation using an independent patient cohort. As we had to exclude patients for whom no suitable CT image was available or segmentation was not possible, our cohort is only of medium size. Furthermore, the dynamic nomogram only estimated survival in China and may not be applicable in other settings. Finally, we were unable to analyze the influence of the histological subtype or gene mutations such as KRAS mutational status in our cohort, as most patients were diagnosed via ascites from abdominal puncture when gene mutation testing was not part of routine diagnostics as well.

## Conclusion

In conclusion, our online prediction model offers a convenient tool to identify patients with unresectable or metastatic PDAC who are at risk of poor survival based on 3D-measured body compositions [low SMI (male < 2474.00 cm^3^/m^2^, female < 1718.00 cm^3^/m^2^), high VFI (male > 2756.00 cm^3^/m^2^, female > 2288.00 cm^3^/m^2^), and high VSR (male > 1.56, female > 0.68)], low TyG-BMI (< 194.50), as well as low prealbumin. Nutritional issues including protein supplementation and resistance training should be taken into account since the time of PDAC diagnosis, and should be integrated into the overall management strategy alongside antineoplastic treatments, aiming to enhance patient survival.

## Data Availability

The raw data supporting the conclusions of this article will be made available by the authors, without undue reservation.

## Footnotes

1
https://pdacdynamicnomogram.shinyapps.io/DynNomapp/
