# Supplementary material for: Synergic value of 3D CT-derived body composition and triglyceride glucose body mass for survival prognostic modeling in unresectable pancreatic cancer
Source: Front Nutr. 2025 Mar 19;12:1499188. doi: 10.3389/fnut.2025.1499188 (PMC11961436; doi:10.3389/fnut.2025.1499188)
Supplement: Supplementary file 1 [file Image_1.pdf]

Supplementary Materials

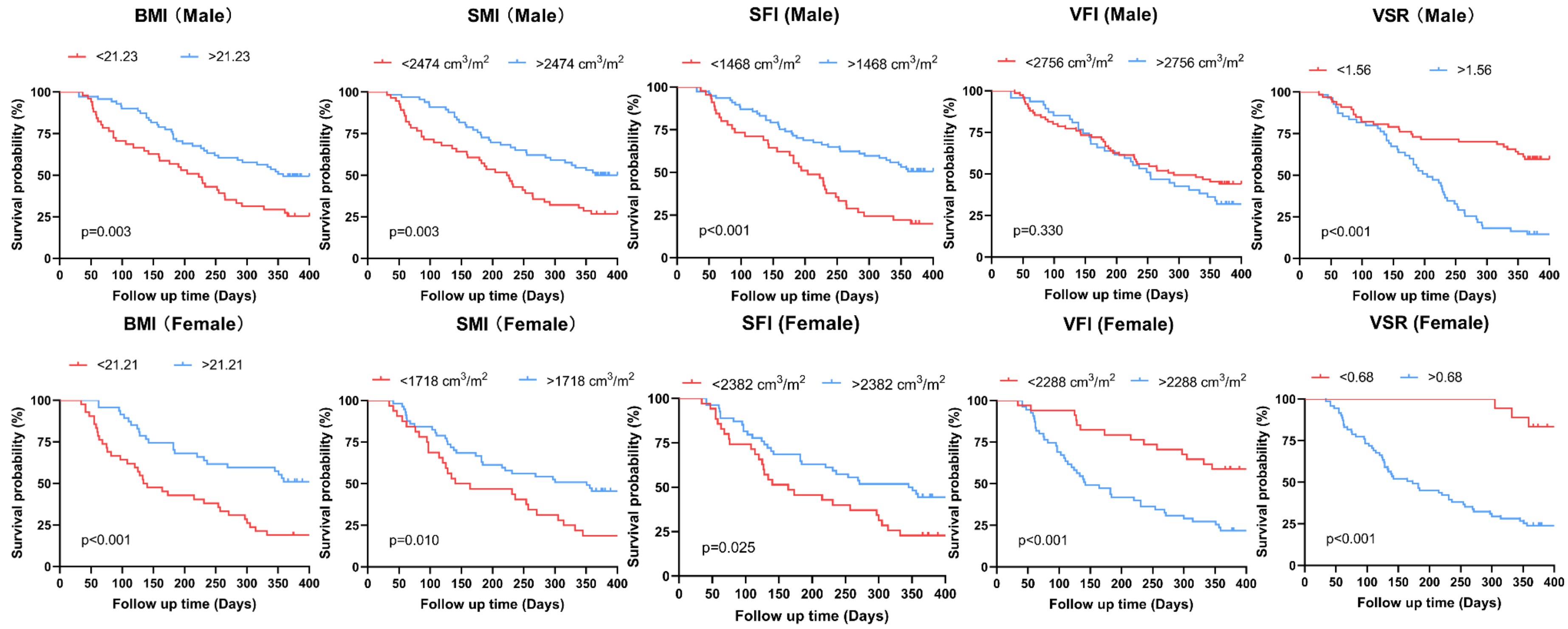

Supplementary Figure 1. Kaplan-Meier estimates of overall survival according to the threshold determined on ROC analysis of BMI, SMI, SFI, VFI, VSR and TyG-BMI for males and females separately.

Abbreviations: BMI, body mass index; SMI, skeletal muscle index; SFI, subcutaneous fat index; VFI, visceral fat index; VSR, visceral to subcutaneous adipose tissue area ratio; TyG-BMI, triglyceride glucose-body mass index.
